# Feasibility of Veno-Venous Extracorporeal Membrane Oxygenation Cannulation via the Neck in a Patient With Spinal Cord Injury Wearing a Halo Vest: A Case Report

**DOI:** 10.7759/cureus.92902

**Published:** 2025-09-22

**Authors:** Ryo Ageishi, Yuichi H, Kazuhiro Masuda, Morio Suzuki, Jun Hamaguchi, Keiki Shimizu

**Affiliations:** 1 Department of Emergency and Critical Care Medicine, ECMO Center, Tokyo Metropolitan Tama Medical Center, Fuchu, JPN; 2 Department of Orthopedic Surgery, Tokyo Metropolitan Tama Medical Center, Fuchu, JPN; 3 Department of Urology, Suzuki Urology Clinic, Nagano, JPN

**Keywords:** halo vest, spinal cord injury, vein cannulation, venoarterial extracorporeal membrane oxygenation (va-ecmo), veno-venous extracorporeal membrane oxygenation (vv-ecmo)

## Abstract

To our knowledge, no previous reports have described the use of veno-venous extracorporeal membrane oxygenation (V-V ECMO) in patients wearing a halo vest. We present a case in which neck cannulation was safely performed through a modified approach. A 38-year-old woman with intractable epilepsy sustained a C5 anterior dislocation and high cervical spinal cord injury after a seizure-related fall. Conservative management was chosen, and sedation with mechanical ventilation was initiated while maintaining a halo vest. On day 20, she developed septic shock complicated by neurogenic shock, leading to cardiac arrest. Extracorporeal cardiopulmonary resuscitation was performed. Despite circulatory improvement, she developed severe respiratory failure and was transitioned to V-V ECMO on day 22. Cannulation was achieved using the right femoral vein and right internal jugular vein for drainage, with the right internal jugular vein serving as the return site. She was successfully weaned from V-V ECMO on day 30 and transferred to a rehabilitation hospital on day 128. This rare case demonstrates that, with appropriate precautions, cervical cannulation for V-V ECMO can be safely performed in patients wearing a halo vest, offering valuable clinical insight for managing similarly complex situations.

## Introduction

Respiratory complications such as atelectasis, pneumonia, and pulmonary edema are among the most frequently observed problems following traumatic spinal cord injury (SCI). These complications significantly contribute to early mortality, with incidence increasing in higher-level injuries [1]. Depending on the level of injury, up to 60% of patients with SCI require invasive mechanical ventilation [2,3]. The halo vest is used to stabilize the cervical and upper thoracic spine [4]. As an external fixation device extending from the head to the trunk, it makes procedures involving the cervical region technically challenging while in place. In certain situations, removal of the halo vest may be necessary to perform interventions; however, this carries the risk of worsening spinal instability.

Even with invasive ventilatory support, patients who develop refractory hypoxemia or in whom lung-protective ventilation cannot be maintained may become candidates for veno-venous extracorporeal membrane oxygenation (V-V ECMO) [5]. Reports describing the use of V-V ECMO in patients with respiratory failure associated with traumatic SCI remain scarce [6]. Furthermore, to our knowledge, no published reports have described V-V ECMO implementation in patients managed with a halo vest. The presence of a halo vest presents significant challenges for cannulation site selection and procedural execution. Although the cervical approach is generally avoided in such cases, we present a rare case in which cervical cannulation was safely performed, resulting in a favorable outcome.

## Case presentation

The patient was a 38-year-old woman with a history of developmental delay and intractable epilepsy associated with Lennox-Gastaut syndrome. Her medications included carbamazepine 600 mg/day, sodium valproate 1400 mg/day, clobazam 30 mg/day, and phenobarbital 60 mg/day. She was 162 cm tall, weighed 62 kg (BMI: 23.6), and resided in a care facility but was ambulatory. Although she experienced daily seizures and had limited verbal communication, she interacted using facial expressions.

Three days before admission, she sustained an occipital head contusion during a seizure and was treated at another hospital. Although she was discharged, impaired consciousness and urinary retention developed, prompting reevaluation one day before admission. A CT scan revealed a dislocated fracture at the C5-6 level, and she was transferred to our hospital.

**Figure 1:**
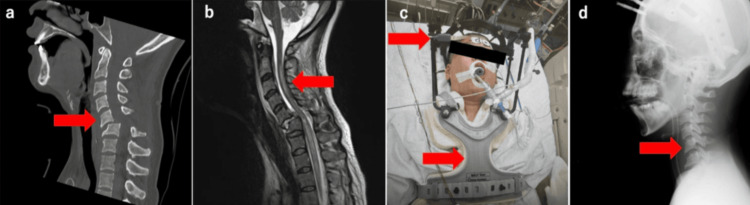
Imaging studies on the day of transport (a) CT scan showing anterior dislocation of C5 (red arrow). (b) T2-weighted MRI showing a high-intensity signal at the C3 level, indicating SCI (red arrow). (c) After endotracheal intubation and application of the halo vest (red arrow). (d) X-ray after manual reduction and halo vest application, showing reduction of the C5 dislocation (red arrow). SCI, spinal cord injury

On arrival, her vital signs were as follows: Glasgow Coma Scale (GCS) E4V2M4, temperature 37.2°C, respiratory rate 18 breaths/min, SpO₂ 96% (room air), pulse rate 80 bpm, and blood pressure 86/55 mmHg. The airway was patent, but chest expansion was poor, and abdominal breathing was noted. Cough strength was also weak, suggesting respiratory muscle paralysis associated with high cervical SCI. A CT scan showed anterior dislocation at C5 (Figure 1a), and a T2-weighted MRI revealed a high-intensity signal extending to the C3 level, indicating cervical SCI (Figure 1b).

Due to narrow pedicles and lateral masses, surgical fixation was deemed anatomically challenging. Given the patient’s neurological status and the difficulty of prolonged bed rest, conservative management was selected. Endotracheal intubation, mechanical ventilation, manual reduction of the dislocation, and application of a halo vest were performed (Figure 1c, 1d). Deep sedation was maintained to prevent redislocation due to seizures, and conservative treatment was planned for eight weeks.

On day 7, the patient developed vomiting, aspiration, and fever and was diagnosed with ventilator-associated pneumonia. Piperacillin/tazobactam was initiated. A tracheostomy was performed on day 9. Although her fever initially improved, it recurred on day 10. Despite ongoing antimicrobial therapy for suspected catheter-related bloodstream infection, her hemodynamics remained unstable. *Bacillus cereus* was isolated from both the central venous catheter tip culture and the blood culture at that time.

On day 20, following endotracheal suctioning, she developed sinus bradycardia (30 bpm) that progressed to pulseless electrical activity. The halo vest was removed, and chest compressions were initiated. After administration of 1 mg epinephrine, spontaneous circulation returned. Due to persistent instability, norepinephrine (1 μg/kg/min) and fluid resuscitation were administered. However, the patient experienced another cardiac arrest, necessitating extracorporeal cardiopulmonary resuscitation with a 21 Fr venous drainage cannula (Capiox^®^, Terumo Corporation, Tokyo, Japan) inserted via the right femoral vein and a 16.5 Fr arterial return cannula (Capiox^®^, Terumo Corporation) inserted via the right femoral artery. After establishing V-A ECMO, ongoing fluid resuscitation was required due to post-cardiac arrest syndrome and septic shock; therefore, vancomycin was added. Cardiac arrest of cardiac origin was excluded.

**Figure 2:**
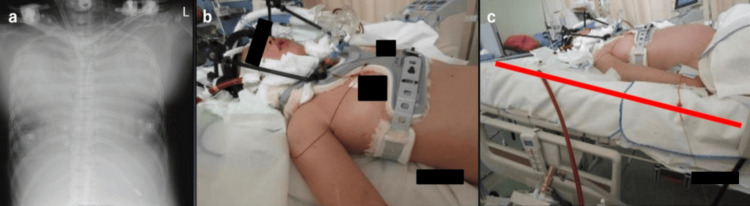
V-V ECMO management under the halo vest (a) Chest X-ray after ECMO initiation showing bilateral infiltrates. A cannula is placed in the right internal jugular vein. (b) Clinical photo showing generalized edema and limited thoracic expansion due to the halo vest. (c) Bed tilted to a 15-30° reverse Trendelenburg position, improving tidal volume (red line). ECMO, extracorporeal membrane oxygenation; V-V ECMO, veno-venous extracorporeal membrane oxygenation

By day 22, her hemodynamics had stabilized (HR: 76 bpm; BP: 106/63 mmHg; no norepinephrine), but she developed pulmonary edema due to excessive fluid administration. Her respiratory status deteriorated, with a P/F ratio of 100 (PEEP: 13 cmH₂O, FIO₂: 1.0), lung compliance of 13 mL/cmH₂O, and diffuse bilateral opacities on chest X-ray. As differential hypoxemia progressed, the configuration was switched from V-A to V-V ECMO (Figure 2a).

**Figure 3:**
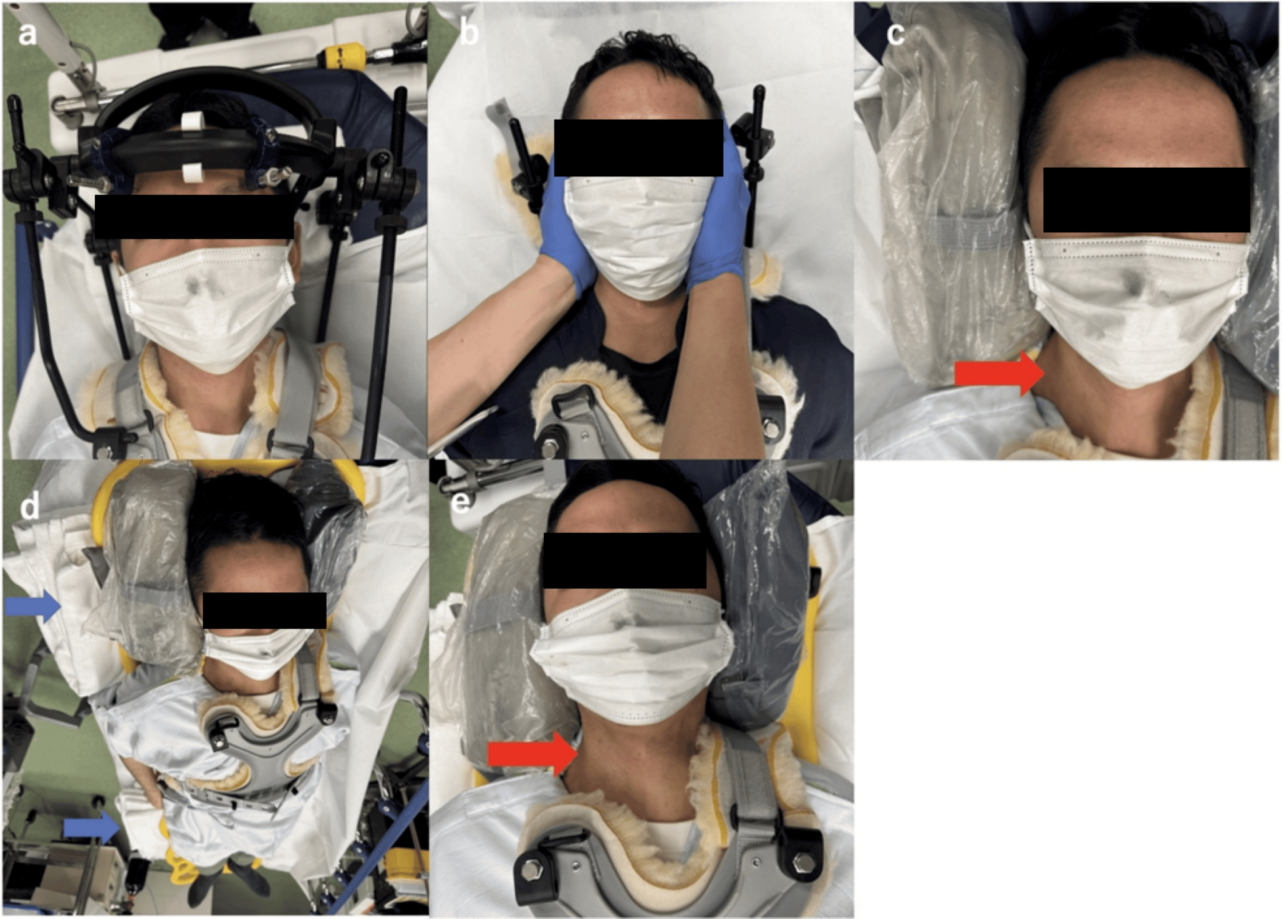
Demonstration of neck cannulation (a) Patient positioned supine with the halo vest in place. (b) The operator stands at the head side and the assistant at the foot side. While the assistant manually stabilizes the head in the midline, the operator carefully removes the halo ring from the head side. (c) Sandbags placed on both sides of the head to maintain midline alignment. Just before disinfecting the puncture site, the assistant releases manual support. After disinfection, cannulation of the right internal jugular vein is performed under standard ultrasound guidance (red arrow). Following initiation of V-V ECMO, the head is again manually stabilized in the midline, the sandbags are removed, and the halo ring is reapplied. (d) Although not used in this case, the right lateral decubitus position may be considered by padding under the back with towels (blue arrow). (e) Use of sandbags to stabilize the head in the midline may further enhance cannulation safety (red arrow). V-V ECMO, veno-venous extracorporeal membrane oxygenation Images were obtained at a later date using a healthy volunteer, not the index patient, and are provided for illustrative purposes only.

The ECMO configuration was converted from V-A to V-V by replacing the arterial return cannula with a 21 Fr venous return cannula (Capiox^®^, Terumo Corporation) inserted into the right internal jugular vein (Figure 3). The ECMO flow rate was 3.2 L/min at 3000 rpm. The ventilator was set to A/C-PC mode with PEEP 10 cmH₂O, PIP 20 cmH₂O, and a respiratory rate of 10 breaths/min, following a lung rest strategy.

Generalized edema, diaphragmatic restriction, and thoracic cage immobility due to the halo vest (Figure 2b) limited tidal volume to 60 mL. By tilting the bed to a reverse Trendelenburg position, tidal volume improved to 150 mL (Figure 2c). Continuous renal replacement therapy (CRRT) was initiated for fluid removal.

**Figure 4:**
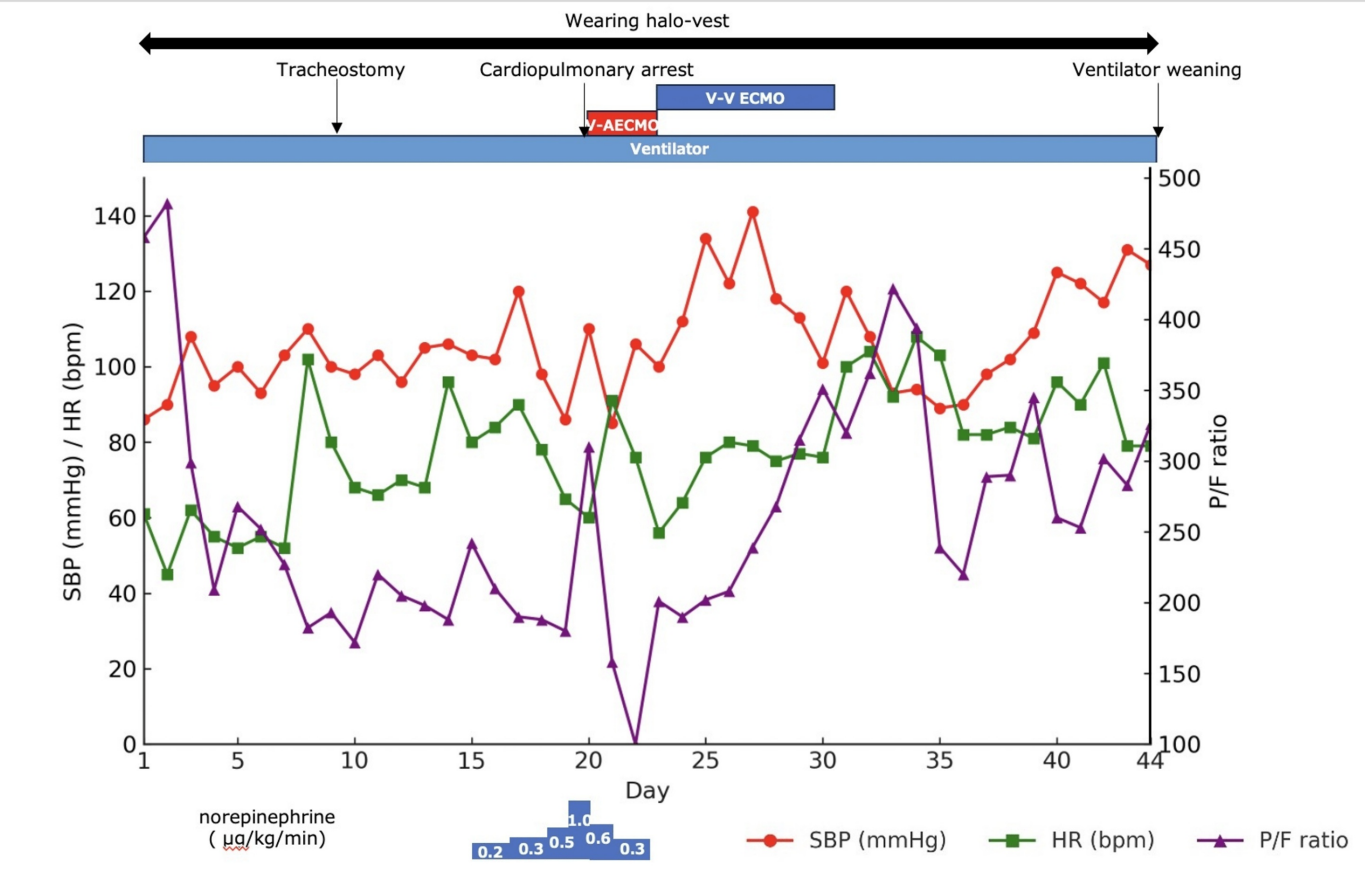
Details of the clinical course HR, heart rate; SBP, systolic blood pressure

She was successfully weaned from V-V ECMO on day 30. Decannulation from the neck was safely performed with the patient in the supine position under the halo vest, and pressure hemostasis was achieved without complications. She was weaned from mechanical ventilation on day 44 and discharged to a rehabilitation facility on day 128. She continued follow-up as an outpatient at our hospital. Details of the clinical course are shown in Figure 4.

## Discussion

This case involved a patient with traumatic cervical SCI who underwent conservative management while wearing a halo vest. During her course, she developed severe respiratory failure requiring V-V ECMO, which was safely established through neck cannulation with careful positioning.

Respiratory complications are common in patients with SCI, particularly those with high cervical involvement. The underlying pathophysiology includes ineffective coughing, impaired secretion clearance, and reduced strength of the diaphragm and thoracic musculature, leading to atelectasis, pneumonia, and hypoventilation [7,8]. In particular, injuries at the C3 level or above result in nearly complete paralysis of respiratory muscles, including diaphragmatic dysfunction, with forced vital capacity reduced to approximately 50% of predicted values [9]. In our patient, abdominal breathing and ineffective ventilation were evident at presentation, necessitating early mechanical ventilatory support. The combined effects of decreased pulmonary compliance due to pulmonary edema from massive fluid resuscitation, diaphragmatic dysfunction resulting from cervical SCI, and reduced thoracic compliance, likely attributable to generalized edema and the adverse effects of the halo vest, culminated in severe respiratory failure requiring the initiation of V-V ECMO [10].

Further deterioration occurred due to fluid overload from septic shock management, leading to decreased lung and thoracic compliance. These factors collectively resulted in severe hypoxemia, necessitating V-V ECMO. Although neck cannulation is often avoided in patients with cervical instability, in this case, the right internal jugular vein was chosen for return cannulation.

When employing two-cannula V-V ECMO configurations, three primary strategies are typically considered: (1) right internal jugular vein drainage with femoral vein return; (2) femoral vein drainage with right internal jugular return; and (3) femoral vein drainage and return. The third option is commonly selected to avoid neck access in patients with cervical instability. In prior reports of V-V ECMO in traumatic cervical SCI, femoral-jugular or dual-lumen cannulas were used, but these were implemented after spinal stabilization surgery [6]. In our case, conservative treatment was chosen due to anatomical and clinical considerations, and cannulation through the internal jugular vein was successfully performed using an adapted technique. Key steps included maintaining the head in a neutral midline position during cannula insertion and providing lateral support with sandbags to avoid neck rotation or extension. This allowed for safe venous access without compromising spinal stability. Given that this technique required neither specialized equipment nor advanced expertise, it should be considered reproducible. Although not used in this case, right lateral decubitus positioning can be considered by padding under the back with towels.

Weaning from V-V ECMO requires addressing the underlying causes of respiratory failure. In our case, interventions targeted pulmonary and thoracic compliance. CRRT was initiated to resolve generalized edema and improve respiratory mechanics. Furthermore, based on prior reports indicating that the reverse Trendelenburg position facilitates diaphragmatic contraction in patients with cervical SCI [11], we utilized bed tilting to promote diaphragmatic function. This resulted in measurable improvement in tidal volume and overall gas exchange.

Because this study is based on a single case, the findings should be interpreted with caution. Additional case reports and series will be essential to further evaluate and validate this approach.

## Conclusions

In patients with traumatic cervical SCI wearing a halo vest, cervical cannulation for V-V ECMO initiation can be performed safely with appropriate modifications. This case highlights the feasibility and safety of neck cannulation and emphasizes the importance of careful positioning and stabilization to minimize risks. Although neck cannulation is often avoided in patients with cervical instability, our experience suggests that it may be a viable alternative in highly selected cases, provided that individualized strategies such as temporary halo vest adjustment and external support are implemented. As this is a single case, caution is warranted in generalizing the findings, and further clinical experience will be needed to validate this approach.
